# Telomere maintenance and dysfunction predict recurrence in paediatric ependymoma

**DOI:** 10.1038/sj.bjc.6604652

**Published:** 2008-09-16

**Authors:** U Tabori, V Wong, J Ma, M Shago, N Alon, J Rutka, E Bouffet, U Bartels, D Malkin, C Hawkins

**Affiliations:** 1 Division of Hematology/Oncology, The Hospital for Sick Children and the University of Toronto, Toronto, ON, Canada; 2 Division of Pathology, Department of Paediatric Laboratory, The Hospital for Sick Children and the University of Toronto, Toronto, ON, Canada; 3 Division of Neurosurgery, The Hospital for Sick Children and the University of Toronto, Toronto, ON, Canada

**Keywords:** telomerase, *γ*H2AX, child, ependymoma, prognosis

## Abstract

We have recently described the enzymatic subunit of telomerase (hTERT) as an important prognostic marker for paediatric ependymoma. Because of the lack of good, representative pre-clinical models for ependymoma, we took advantage of our large cohort of ependymoma patients, some with multiple recurrences, to investigate telomere biology in these tumours. Our cohort consisted of 133 ependymomas from 83 paediatric patients and included 31 patients with recurrences. Clinical outcome was measured as overall survival, progression-free survival and response to therapy. In all 133 tumours, hTERT expression correlated with proliferative markers, including MIB-1 index (*P*<0.0001) and mitotic index (*P*=0.005), as well as overall tumour grade (*P*=0.001), but not with other markers of anaplasia. There was no correlation between telomere length and hTERT expression or survival. Surprisingly, prior radiation or chemotherapy neither induced sustained DNA damage nor affected telomere maintenance in recurrent tumours. There was an inverse correlation between hTERT expression and telomere dysfunction as measured by *γ*H2AX expression (*P*=0.016). Combining *γ*H2AX and hTERT expressions could segregate tumours into three different survival groups (log rank, *P*<0.0001) such that those patients whose tumours expressed hTERT and showed no evidence of DNA damage had the worst outcome. This study emphasises the importance of telomere biology as a prognostic tool and telomerase inhibition as a therapeutic target for paediatric ependymoma. Furthermore, we have demonstrated that analysing tumours as they progress *in vivo* is a viable approach to studying tumour biology in humans.

Ependymoma is the third most common paediatric brain tumour. Although there is substantial clinical experience with ependymoma treatment, tumour behaviour is extremely variable, ranging from an aggressive course to prolonged survival with multiple relapses (Bouffet *et al*, 1998). Although recent attempts at standardising grading criteria for ependymoma (Tihan *et al*, 2008) have improved reproducibility, there is still a large group of tumours for which assigning a grade is difficult. Furthermore, biological prognostic factors for this disease have been lacking (Packer, 2005; Rickert and Paulus, 2005). To address this problem, we recently reported that the expression of hTERT, the enzymatic subunit of human telomerase, is the single most important predictor of outcome among known pathological and clinical risk factors at diagnosis (Tabori *et al*, 2006a).

The telomerase complex is integral for telomere maintenance. Telomeres are unique structures at the ends of chromosomes whose function is to form ‘caps’ that prevent DNA ends being recognised as DNA breaks, which would be catastrophic to normal cells. Telomeres of proliferating cells become shorter until a critical phase at which they become dysfunctional, resulting in higher genomic instability or irreversible growth arrest. Telomere maintenance is one of the hallmarks of cancer and this is believed to allow their continued proliferation (Hanahan and Weinberg, 2000). Most cancer cells maintain their telomeres by reactivating hTERT. Others do so by a poorly understood mechanism called alternative lengthening of telomeres (ALT). Although clearly telomerase has a critical function in ependymoma behaviour, currently, there are no data regarding which functions of telomerase are underlying its association with aggressiveness in paediatric ependymoma.

Our initial finding of telomerase expression in ependymomas highlights the need for improved understanding of telomere biology for the development of better prognostic markers and new therapies for these devastating tumours. Unfortunately, the ability to examine functional biological questions in ependymomas has been hampered by the lack of available cell lines and other preclinical models. Therefore, in this study, we took advantage of our large cohort of ependymoma patients, some with multiple recurrences, to investigate telomere biology in these tumours.

Here, we report the biology of telomere maintenance, its prognostic significance and the effect on response to therapy in paediatric ependymoma *in vivo*. Furthermore, the general methodology described here may be used as an alternative approach to studying biological processes in tumours where cell lines are not available.

## Materials and methods

### Patients

Patients operated on at the Hospital for Sick Children (SickKids, Toronto, Canada) between 1986 and 2004 with a pathologic diagnosis of intracranial ependymoma were identified retrospectively through the pathology and neuro-oncology databases. Patients with spinal ependymomas were excluded. In total, 133 tumours from 83 patients were identified and the pathology reviewed. This included the cohort of our previous study (Tabori *et al*, 2006a) with additional patients, and complete data of recurrence and repeated surgeries/treatments. Formalin-fixed, paraffin-embedded material was available for all 133 specimens and was used for tissue microarray construction (see below). For a subset of 26 of these specimens, matched fresh-frozen material was available for measuring telomerase enzymatic activity and telomere length (see below).

Thirty-one patients died of their disease. Fifty-two survivors were actively observed; of these, 9 patients were lost to follow-up and, for this analysis, were censored at the time of their last clinic visit. The study had prior approval from the Research Ethics Board at SickKids. All data were anonymised prior to publication.

### Clinical data

Clinical data collected included age and metastatic disease status at presentation, sex, the extent of surgical resection, chemotherapy use (including protocol and drugs used), radiotherapy use (including dose and field), progression-free survival (PFS) and overall survival (OS). For survival analysis per tumour sampled, progression was the primary end point. Complete metastatic work-up data, including spinal MRI and CSF cytology, were available for all patients. The extent of surgical resection was recorded as gross total resection (GTR) if no tumour was apparent, as described in the surgical report or on postoperative magnetic resonance imaging. The latter imaging technique was used for all patients. Subtotal resection (STR) was defined as less than 50% residual tumour, and partial resection (PR) as more than 50% residual tumour. Tumours that were only biopsied were recorded as such. Total radiation dose to the tumour bed and craniospinal axis were recorded separately. Chemotherapy regimens and times were recorded for initial diagnosis and recurrences. A summary of the clinical data and tumour hTERT status by resection number is shown in Table 1.

### Morphologic evaluation

A number of histologic features are used to define an ependymoma as either WHO grade 2 or 3, although no definitive criteria have been universally accepted. We analysed multiple morphologic features that have been suggested to indicate tumour grade including: mitotic index, MIB-1 (Ki67) proliferative index, hypercellular foci, nuclear pleomorphism, perivascular pseudorosettes, vascular endothelial proliferation and necrosis. Quantification of the MIB-1 index was measured as the number of positive cells expressed as a percentage of total tumour cells in the three high-power fields with greatest positivity (Grotzer *et al*, 2001). This analysis was carried out using Simple PCI software (Nikon Canada Inc., Mississauga, ON, Canada). Mitotic index was counted by two observers blinded to clinical data and expressed as number per 10 high-power fields. Each of the other features was evaluated by two observers blinded to clinical data and recorded as present or absent. For the analysis of grade for this study, the grade assigned by the original pathologist at the time of case sign out was used (Tables 1 and 2).

### Ependymoma tissue array construction

Tissue arrays were prepared as described earlier by our group (Ray *et al*, 2004; Tabori *et al*, 2006a). Briefly, for each patient, all pathologic blocks and corresponding slides were obtained and reviewed by a neuropathologist (CH) for diagnostic accuracy and tissue adequacy. Representative tumour areas were identified, and three 1-mm cores were obtained from each tumour, providing a sampling accuracy of at least 95% (Hoos and Cordon-Cardo, 2001). A variety of tissues, including the liver, ependyma, choroid plexus, neuroblastoma, and breast cancer, were included around the periphery of each array to serve as internal controls.

### Immunohistochemistry

Sections of 5 *μ*m were cut from the tissue microarray and mounted on positively charged microscope slides. Tissue sections were then baked overnight at 60°C, dewaxed in xylene and hydrated with distilled water through decreasing concentrations of alcohol. Immunohistochemistry of hTERT (clone 44F12; Novocastra, Newcastle upon Tyne, UK) (Yan *et al*, 2004) and *γ*H2AX (clone JBW301, Millipore/Upstate, Mississauga, ON, Canada) was performed manually at dilutions of 1 : 25 and 1 : 1000, respectively, incubated overnight at 4°C and immunodetected using the Vector Elite avidin–biotin complex method detection system (Vector Laboratories, Burlingame, CA, USA). MIB-1 (Ki-67 antigen, DakoCytomation, Dako Canada Inc., Mississauga, ON, Canada), at a dilution of 1 : 20, was performed on the Ventana NEXES auto-immunostainer (Ventana Medical Systems, Tucson, AZ, USA), with a closed avidin–biotin complex method system using the 3,3-diaminobenzidine Ventana Detection System. All tissue sections were treated with heat-induced epitope retrieval and blocked for endogenous peroxidase and biotin. The counterstain of preference was haematoxylin. Appropriate positive and negative controls were also tested in parallel. Conventional sections from paraffin blocks were used for samples that were of poor quality and for tumours that were resected after the construction of the tissue array.

### Immunohistochemical grading

Immunohistochemical staining for hTERT and *γ*H2AX (nuclear) was reviewed and graded for both strength (0, none; 1, weak; and 2, strong) and distribution (<25, 25–50 and >50% of tumour cells) as described earlier by us (Tabori *et al*, 2006a). The reviewers were blinded to clinical patient data at the time of grading. Only tumours with strong (grade 2) nuclear staining in more than 25% of tumour cells were considered to be positive for hTERT and *γ*H2AX expressions. This definition of hTERT positivity correlated well with telomerase activity in our previous study.

### Telomerase activity assay

Tissue of 50 mg was homogenised with CHAPS lysis buffer. The protein concentrations of the samples were determined and equal quantities used for the assay. Samples were analysed with TRAPeze kit (Millipore (Canada) Ltd., Etobicoke, Ontario, Canada) reagents according to the manufacturer's instructions using 30 min of telomerase extension at 30°C, and inactivating telomerase at 85°C for 10 min. The extension products were amplified by 35 PCR cycles (94°C for 30 s, 56°C for 30 s and 72°C for 60 s). A standard batch of HeLa cells was used as a positive control and lysis buffer was used as negative control for each run. Polymerase chain reaction samples (20 AL) were analysed on a 10% non-denaturing acrylamide gel in 0.5 Tris–borate EDTA at 200 V for 4 h. Gels were stained using Syber-Gold and scanned.

### Terminal restriction fragment assay

Telomere lengths were determined by a terminal restriction fragment (TRF) kit (Roche Diagnostics, Mannheim, Germany). Briefly, extracted DNA samples (1–2 *μ*g of tumour DNA) were digested with the restriction enzymes *Rsa*I and *Hin*fI at 37°C for 2 h and run on 0.8% agarose gels at 10 V for 18 h. A biotinylated *γ*-DNA molecular weight marker was used as DNA length standard. High- and low-molecular-weight DNAs were run as positive controls. The DNA samples were depurinated in 0.25 mol l^−1^ of HCl, denatured in 0.4 mol l^−1^ of NaOH/3 mol l^−1^ of NaCl, and transferred to a positively charged nylon membrane Hybond-N (Amersham Pharmacia Biotech, Little Chalfont, UK) by capillary blotting over 12 h. The membrane was washed in saline–sodium citrate buffer. The blot was hybridised with a (TTAGGG)^3^ telomere probe at 42°C for 3 h and washed in 2 × SSC/0.1% SDS. Chemiluminescent detection was performed according to the Detection Kit (Roche Diagnostics, Basel, Switzerland). Detection was carried out on an X-ray Hyperfilm ECL. To address the issue of tissue heterogeneity, mean TRF lengths were calculated as *ε*(OD_*i*_)/*ε*(OD_*i*_/L_*i*_). The final number represents the mean molecular size of 36 equal to intervals of the telomeric smears in the range of 2–20 kb as defined by the DNA length standard. OD_*i*_ reflects the measured intensity of luminescence in each of the 35 intervals. As reported in the literature, TRF lengths were recorded as telomere lengths.

### Telomere fluorescence *in situ* hybridisation/*γ*H2AX co-staining

Formalin-fixed, paraffin-embedded sections of 5 *μ*m thickness were dewaxed in xylene followed by rehydration in alcohol series and allowed to air dry. Antigen retrieval was carried out using a standard citrate buffer/pressure cooker method for 22 min. Slides were allowed to cool to room temperature, followed by a brief rinse in water and two washes in 2 × SSC for 5 min. After dehydration in alcohol series, cy3-conjugated telomeric PNA probes (Dako, Mississauga, ON, Canada) was applied and co-denatured at 80°C for 5 min followed by hybridisation at room temperature for 1 h. Slides were washed following the manufacturer's protocol. Slides were then blocked with donkey serum (1 : 50) at room temperature for 3 h followed by the addition of monoclonal mouse anti-*γ*H2AX antibody (1 : 1000, Upstate Cell Signaling, Temecula, CA, USA) for an overnight incubation at 4°C. Slides were washed four times in BSA/PBS (0.65 g l^−1^), followed by the addition of FITC-conjugated donkey anti-mouse secondary antibody (1 : 100) for 30 min incubation at room temperature. Slides were washed four times in BSA/PBS followed by alcohol dehydration. Slides were counterstained with DAPI and viewed under a Nikon Eclipse E400 fluorescent microscope with appropriate filters (Nikon Instruments, Toronto, ON, Canada). Digital images were captured with a Nikon DXM1200F camera and analysed using open source software ImageJ (http://rsb.info.nih.gov/ij/).

### Statistical analysis

For each biological and clinical marker, PFS and OS were estimated using the Kaplan–Meier method, and significance testing (*α*=0.05) performed on the basis of the log-rank test. Multivariate analysis was performed using multivariate Cox proportional hazards models and significance testing (*α*=0.05) performed on the basis of the Wald test. Correlation between hTERT expression and other parameters was assessed using the Pearson's *χ*^2^, with *P*<0.05 considered a significant correlation.

## Results

### hTERT expression correlates with proliferative but not anaplastic markers in ependymomas

We measured multiple markers of proliferation and anaplasia commonly used in ependymomas (Table 2). We then examined whether there was a correlation between each of these parameters and hTERT expression, as well as their ability to predict PFS (Table 3). Of the morphologic parameters tested, only MIB-1 proliferative index >12% was associated with PFS. hTERT expression correlated strongly with MIB-1 (Ki67) proliferative index (Pearson's *χ*^2^ *P*<0.0001), mitotic index (Pearson's *χ*^2^ *P*=0.005) and tumour grade (Pearson's *χ*^2^ *P*=0.001). Grade 2 ependymomas (63%) were hTERT(−) as compared with only 11% of grade 3 ependymomas (*P*=0.002). Interestingly, hTERT expression did not correlate with other morphologic markers of tumour anaplasia – marked hypercellularity, nuclear pleomorphism, loss of perivascular pseudorosetting, the presence of necrosis or vascular proliferation. These findings are in keeping with a specific role for telomerase in allowing continued cellular proliferation in tumorigenesis.

### Telomerase protects telomeres regardless of telomere length

**Figure 1:**
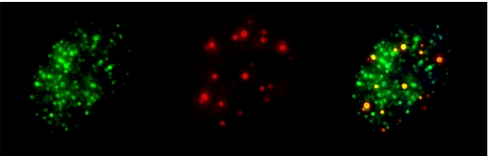
Co-staining for telomeres and *γ*H2AX. Representative nucleus showing double labelling with the *γ*H2AX antibody (green, left panel) and telomeric FISH probe (red, middle panel). The merged view (right panel) shows yellow dots demonstrating DNA damage (*γ*H2AX positivity) at the telomeres.

To determine the correlation between hTERT expression and telomere status and function in ependymomas, we tested for telomerase activity, telomere length and DNA damage. The former two studies were performed on a subset of samples for which frozen tissue was available (26 samples). The presence of DNA damage was assessed by the expression of phosphorylated H2AX (*γ*H2AX), a protein that is rapidly recruited to the sites of DNA double-stranded breaks or telomere damage (d'Adda di Fagagna *et al*, 2003; Takai *et al*, 2003). Co-labeling of telomere ends (telomeric fluorescence *in situ* hybridisation probes) and *γ*H2AX (immunohistochemistry) suggests that, at least some of, the *γ*H2AX expression is related to telomere damage in these tumours (Figure 1).

**Figure 2:**
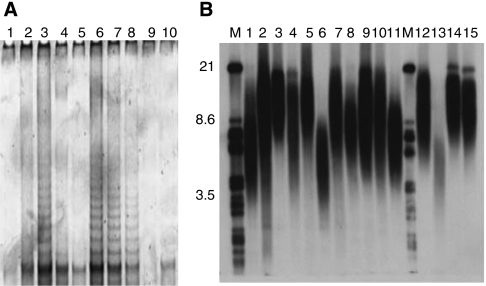
Telomere length and telomerase activity in our cohort. (**A**) Telomerase activity (TRAP assay; lane 1: buffer, lane 2: HeLa control, lane 3: heat-inactivated control, lanes 4–10: tumour samples.) (**B**) Telomere length (TRF assay; M represents molecular marker, lanes 1–15: tumour samples.) was assessed in 26 samples. There was high correlation between telomerase activity and hTERT expression (*P*<0.0001). We did not find evidence of alternative lengthening of telomeres in this cohort.

For a subgroup of 26 patients, which did not differ in terms of clinical features from the overall group, frozen tissue was available for telomere length analysis. In contrast to our study of low-grade astrocytomas, which were telomerase negative and had long telomeres, ependymomas showed a marked variability in telomere length (mean 6.5 kb, range 3.6–9.1 kb) (Figure 2). We did not detect the abnormal heterogeneous TRF signal that is found in ALT (White *et al*, 2001) in this cohort. There was a strong correlation between hTERT expression and enzymatic activity (Pearson's *χ*^2^ *P*<0.0001, Table 3). Interestingly, there was no association between the absence of hTERT expression and shorter telomeres (<6 kb) (Pearson's *χ*^2^ *P*=0.56). Moreover, short telomeres were not associated with evidence of DNA damage (*γ*H2AX expression) or with PFS (Table 3).

In contrast, there was an inverse correlation between *γ*H2AX (DNA/telomere damage) and hTERT expressions (Pearson's *χ*^2^ *P*=0.011). Of 67 hTERT(+) tumours, 52 (78%) lacked *γ*H2AX expression as compared with 20 (54%) of 37 hTERT(−) tumours, suggesting a protective effect of hTERT against increased DNA/telomere damage. This was true regardless of telomere length, indicating that hTERT could protect even short telomeres from becoming dysfunctional. Conversely, in the absence of hTERT expression, 100% (four out of four) of ependymomas with long telomeres exhibited *γ*H2AX and did not progress clinically. These observations suggest that in the absence of telomerase, DNA damage and tumour growth arrest can occur even before significant telomere shortening.

### Telomerase in recurrent tumours

To overcome the lack of cell lines or other preclinical models and to assess aspects of telomere maintenance over time *in vivo*, we analysed patients who had had recurrent resections due to tumour progression. Clinical data and hTERT status by resection number are summarised in Table 1. Thirty-seven patients had at least 2 resections and 12 had at least 3 resections. Of 14 grade 2 tumours, 7 had changed to grade 3 at relapse. Of the seven patients who progressed from grade 2 to grade 3, five were hTERT(+) both at initial presentation and recurrence and 2 were hTERT(−) at initial presentation and hTERT(+) at recurrence. None of 23 grade 3 tumours regressed to grade 2. Of the grade 3 ependymomas, six were hTERT(−) at initial presentation and two of these became hTERT(+) with recurrence. Five of 10 hTERT(−) tumours recurred as hTERT(+). Only 1 of 22 hTERT(+) tumours recurred as hTERT(−). However, this tumour recurred again and became hTERT(+), indicating a likely sampling artefact at the second resection.

Taken together, these findings support the concept that tumours tend to evolve towards a more aggressive biological phenotype at relapse. However, interestingly tumours that continued to lack hTERT expression had a better OS even at recurrence (log rank *P*=0.05). The mean OS was 8.6±2.2 years in the patients who were hTERT(−) at relapse as compared with 1.8±0.37 years in the patients who were hTERT(+) at relapse. None of the other factors (MIB-1, number of mitoses, nuclear pleomorphism, necrosis, hypercellularity or vascular proliferation) were predictive of outcome at recurrence or showed significant differences between consecutive samples. To our knowledge, hTERT is the first described prognostic factor for recurrent ependymomas (Figure 2).

### Effect of radiation and chemotherapy on telomere maintenance

To assess resistance to prior therapy, we analysed *γ*H2AX as a marker of DNA damage in recurrent tumours from patients who had undergone radiation and/or chemotherapy between their first and second resections. The average duration between radiation and re-resection was 3.1 years (s.d.: 1.7), thus short-term effects of radiation could not be assessed. Of 18 irradiated tumours, 15 (83%) were negative for *γ*H2AX. Non-irradiated tumours had the same rate of *γ*H2AX negativity (84%) regardless of time from therapy. Tumours from 10 of 13 (77%) patients treated with chemotherapy exhibited the same pattern of negative *γ*H2AX at the next resection, indicating a lack of prolonged DNA damage response to chemotherapy in recurrent ependymomas. Only one of 15 (7%) and none of 17 (0%) tumours exhibited reduction in hTERT expression after radiotherapy and chemotherapy, respectively, suggesting no long-term effects of treatment on hTERT status.

### Combining telomerase expression and tumour DNA/telomere damage stratifies patients into three prognostic groups

Previously, we reported that hTERT expression predicts survival in paediatric ependymoma at first resection (Tabori *et al*, 2006a). To determine whether proliferative markers that correlate with hTERT expression could predict outcome, we analysed each marker separately. Of the proliferation and differentiation markers tested (Table 3), only MIB-1 was associated with improved PFS at first surgery. Using the median as a cutoff between high (>12%) and low (⩽12%) MIB-1 proliferative index, we found that MIB-1>12% was predictive of a reduced PFS (*P*=0.021) and approached significance in predicting a poorer OS (*P*=0.084) for patients at their initial surgical resection. MIB-1 failed to predict PFS in recurrent tumours (*P*=0.1) and was not independent of hTERT status in predicting outcome in multivariate analysis.

We next examined whether *γ*H2AX expression (i.e., DNA/telomere damage) influenced survival in the current cohort. At first surgical resection, *γ*H2AX expression was predictive of a better PFS (log rank, *P*=0.007) and better OS (log rank, *P*=0.027) (Table 4). Progression-free survival was 45 and 19% in 115 tumours expressing and lacking *γ*H2AX, respectively (*P*=0.007). *γ*H2AX was not independent of hTERT in predicting survival on multivariate analysis (Table 4), but by combining *γ*H2AX and hTERT expressions, we could stratify patients into three statistically significant groups (Figure 4). Patients with tumours who lacked telomerase and showed dysfunctional telomeres (hTERT(−)/*γ*H2AX(+)) had 5-year PFS and OS of 69+15% and 100%, respectively; whereas patients with tumours who maintained their telomeres (hTERT(+)/*γ*H2AX(−)) had 5-year PFS and OS of 17+8% and 22+9%, respectively (*P*<0.0001). After 8 years, only 5% of the latter group had survived (Figure 4). Patients with hTERT(+)/*γ*H2AX(+) and hTERT(−)/*γ*H2AX(−) tumours exhibited intermediate outcomes.

## Discussion

Ependymomas are glial tumours with erratic clinical behaviour. Some tumours behave like low-grade gliomas with prolonged remissions, whereas others progress relentlessly in a manner similar to high-grade gliomas. The biological relevance of telomere maintenance in paediatric ependymomas has, until recently, been poorly understood. While exploring improved prognostication and the identification of potential targets for new therapies, we recently reported that hTERT (the catalytic subunit of telomerase) expression is an excellent predictor of survival in newly diagnosed paediatric ependymomas (Tabori *et al*, 2006a). hTERT expression was found in 58% of ependymomas and conferred a worse prognosis. This finding led to a number of relevant questions concerning the role of telomere biology in paediatric ependymoma, some of which are addressed in this study.

Telomere maintenance is one of the hallmarks of cancer and this is believed to allow their continued proliferation (Hanahan and Weinberg, 2000). Another function of telomerase is to prevent DNA ends being recognised as DNA breaks, which would be catastrophic to normal cells. Thus, we hypothesised that hTERT(+) tumours would show more proliferative potential, less DNA damage and longer telomeres. Our data support a role for telomerase in ependymomas in allowing proliferation as hTERT expression correlated with both increased mitotic index and MIB-1 proliferative index in our ependymoma cohort (Table 3). This is important as a variety of studies (De Semir *et al*, 2007; Jackson *et al*, 2007) have suggested other roles for telomerase in proliferating cells.

Furthermore, our data support a role for telomerase in protecting cells from DNA damage by demonstrating an inverse correlation between hTERT and *γ*H2AX expressions (Table 3). However, in contrast to our hypothesis, we found no correlation between telomere length and hTERT expression or evidence of DNA damage. This observation suggests the importance of telomerase in protecting telomeres even at very short lengths. However, telomere homoeostasis is regulated by multiple and complex protein interactions, including the proteins of the shelterin complex, thus other proteins may also have a function.

There was no correlation between hTERT or *γ*H2AX status and prior chemotherapy or radiotherapy, suggesting no long-term effects of treatment on the expression of these proteins. This is also in keeping with an intact p53 pathway in ependymomas as has been suggested by several other studies (Fink *et al*, 1996; Gaspar *et al*, 2006).

Within the glial neoplasms, low-grade gliomas lack telomerase activity and ALT, and thus fail to maintain their telomeres (Chong *et al*, 1998; Tabori *et al*, 2006b). This results in progressive telomere attrition and may be associated with the unique tendency of paediatric low-grade gliomas to undergo growth arrest (Tabori *et al*, 2006b) and their associated excellent survival. On the other hand, telomerase is actively expressed in most high-grade astrocytomas and embryonal brain tumours (Falchetti *et al*, 2002; Hakin-Smith *et al*, 2003), which may contribute to their worse outcome. Telomere maintenance also seems to have a function in prognosis in adult high-grade gliomas, in which 15% of tumours display ALT and this confers a better prognosis (Hakin-Smith *et al*, 2003). We examined this in our current study and found no evidence of ALT in any of the 26 samples available to be tested. This suggests that ependymomas rely predominantly on telomerase activity to maintain their telomeres and may explain our findings regarding telomere length.

**Figure 3:**
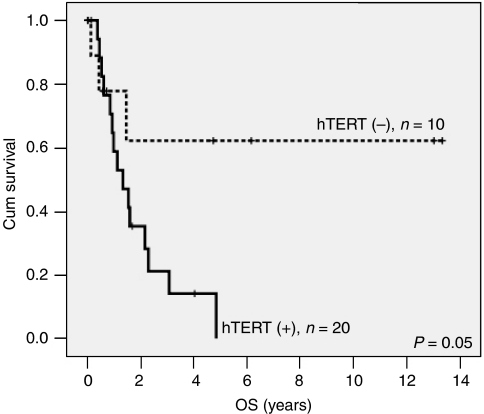
hTERT expression predicts survival in recurrent ependymoma. Overall survival was assessed for ependymomas at first recurrence (*n*=30). There were no long-term survivors in the hTERT(+) tumours (*P*=0.05).

**Figure 4:**
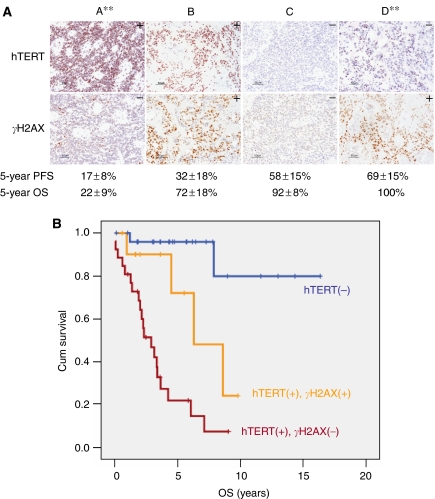
hTERT and *γ*H2AX expressions as prognostic markers in paediatric ependymomas. The addition of *γ*H2AX expression is able to further subdivide patients into prognostic groups. Group A: hTERT(+)/*γ*H2AX(−); Group B: hTERT(+)/*γ*H2AX(+); Group C: hTERT(−)/*γ*H2AX(−); and Group D: hTERT(−)/*γ*H2AX(+) (**A**). Note that patients with hTERT(−) and *γ*H2AX(+) tumours are all alive while there is only 22% 5-year survival for those with hTERT(+), *γ*H2AX(−) tumours. ^**^P<0.001 (Group A *vs* Group D). (**B**) The Kaplan–Meier analysis showing that those patients with *γ*H2AX(+) tumours faired better even when hTERT continued to be expressed. OS, overall survival; PFS, progression-free survival. Values represent mean±s.e. of the mean.

The biological importance of our findings is supported by the prognostic significance conferred by the demonstration of telomere maintenance (hTERT expression) and lack of telomere dysfunction (*γ*H2AX negativity) in paediatric ependymomas (Figures 3 and 4). The striking difference in PFS between morphologically similar tumours, which lack hTERT expression and show telomere dysfunction (*γ*H2AX positivity) on the one hand, and tumours that express hTERT and have functional telomeres (*γ*H2AX(−)) on the other, may allow for a reevaluation of tailored management for these patients. Development of a biologically rational therapy is especially important because most of these patients are young children for whom exposure of the developing brain to conventional high-dose radiation and chemotherapy often leads to devastating short- and long-term neurocognitive sequelae. A possible model may include reduction or delaying chemoradiation for patients in Group D (Figure 4), whereas introducing telomerase inhibitors to Group A patients.

It is also important to note that the ability to assess tumour progression *in vivo* over a period of several years and following different treatment regimens offers an opportunity for a more accurate view of the real biological mechanisms that govern tumour progression. This is particularly relevant as ependymomas lack relevant preclinical models and given the recent challenges to the reliability of cell lines to mimic *in vivo* tumour behaviour (Lee *et al*, 2006).

In summary, this study adds new dimensions to our understanding and utilisation of telomere biology for prognostic and predictive purposes in paediatric ependymomas. Our results are particularly timely, as telomerase inhibitors are currently under evaluation in adult phase I clinical trials, and they may thus offer an attractive option for the management of children with ependymomas as well.

## Figures and Tables

**Table 1:** Clinical features and hTERT status by resection number

	***N* (%)**		
**Characteristic**	**First resection**	**Second resection**	**Third resection**
*Age* >*3 years*			
Yes	46 (61)	22 (55)	8 (67)
No	29 (39)	18 (45)	4 (33)
			
*Sex*			
Male	41 (55)	21 (53)	4 (33)
Female	34 (45)	19 (47)	8 (67)
			
*Tumour location*			
Supratentorial	21 (28)	8 (21)	4 (33)
Infratentorial	52 (72)	31 (79)	8 (67)
			
*WHO grade*			
2	29 (39)	10 (29)	1 (9)
3	46 (61)	25 (71)	10 (91)
			
Metastatic*			
Yes	10 (17)	21 (78)	3 (75)
No	49 (83)	6 (22)	1 (25)
			
*Resection*			
GTR	32 (43)	14 (37)	2 (18)
Subtotal	38 (52)	20 (53)	9 (82)
Partial	1 (1)	2 (5)	0 (0)
Biopsy	3 (4)	2 (5)	0 (0)
			
*Radiation*			
Yes − Focal	28 (38)	6 (15)	0 (0)
Yes − Focal+CS	19 (26)	6 (15)	1 (9)
No	26 (36)	28 (70)	11 (91)
			
*Chemotherapy*			
Yes	38 (53)	17 (57)	6 (50)
No	34 (47)	13 (43)	6 (50)
			
*hTERT*			
Positive	43 (61)	21 (68)	7 (78)
Negative	28 (39)	10 (32)	2 (22)

* Metastatic disease was defined as either the presence of malignant cells on cerebrospinal fluid cytology or definite radiographic evidence of spread prior to the onset of chemo- or radiotherapy.

**Table 2:** Summary of tumour assays done in the study

**Criteria**	**Tumours analysed**	**Parameter measured**	**%**	**Median (range)**
WHO grade	133	Grade 3	64	
		Grade 2	36	
*Proliferative markers*				
MIB1	109	% Positive		12 (0–69)
Mitoses	94	No. per 10 HPFs		5 (0–76)
				
*Markers of anaplasia*				
Loss of pseudorosetting	93	Yes	21	
Nuclear atypia	93	Moderate/severe	58	
Foci of necrosis	94	Yes	69	
Vascular proliferation	94	Yes	46	
Hypercellularity	92	Yes	45	
				
hTERT	113	Positive	62	
*γ*H2AX	115	Positive	32	
TRF	26	Telomere length (kb)		6.5 (3.6–9.1)
TRAP	26	Positive	73	

Measurements and grading of each assay are detailed in the Materials and Methods section.

**Table 3:** Correlation of pathological markers with telomere maintenance factors and progression-free survival

**Parameter**	**hTERT(+) *P*-value (*χ*^2^)**	***γ*H2AX(−) *P*-value (*χ*^2^)**	**PFS univariate *P*-value (log rank)**
MIB1>12%	<0.0001	0.27	0.02
Tumour grade 3	0.001	0.002	0.018
Hypercellularity	0.075	0.03	0.31
Mitoses >5/10 HPFs	0.005	0.006	0.39
Loss of pseudorosettes	0.29	0.8	0.42
Foci of necrosis	0.8	0.3	0.87
Vascular proliferation	0.72	0.09	0.28
Nuclear atypia	0.74	0.31	0.6
*γ*H2AX-negative	0.016	—	0.007
Telomere length <6.5	0.6	0.4	0.44
TRAP-positive	<0.0001	0.6	0.04

**Table 4:** Multivariate analyses of clinical and biological markers

**Parameter**	**PFS univariate *P*-value (log rank)**	**PFS multivariate *P*-value (Wald)**	**OS univariate *P*-value (log rank)**	**OS multivariate *P*-value (Wald)**
Extent of resection	0.011	0.021	0.091	0.337
Age (years)	0.012	0.028	0.021	0.22
Metastatic disease	0.02	0.886	0.066	0.372
WHO grade	0.018	0.128	0.016	0.301
*γ*H2AX	0.007	0.133	0.027	0.247
hTERT	0.001	0.075	<0.0001	0.002
MIB1	0.021	0.122	0.084	0.472
